# Measuring Compliance and Perceptions of an Anterior Cruciate Ligament (ACL) Injury Prevention Programme: Insights From Female University Athletes

**DOI:** 10.7759/cureus.88368

**Published:** 2025-07-20

**Authors:** Elizabeth Mainwaring, Georgia Tooth, Benjamin D Gompels, Jai Pantling, Ava Machesney, Georgia Weeks, Niel Kang, Stephen McDonnell

**Affiliations:** 1 Division of Trauma and Orthopaedics, University of Cambridge, Cambridge, GBR; 2 Department of Trauma and Orthopaedics, Addenbrooke's Hospital, Cambridge University Hospitals NHS Foundation Trust, Cambridge, GBR

**Keywords:** acl injury, anterior cruciate ligament (acl), female athlete, knee injuries, trauma and injury prevention

## Abstract

Objectives

This pilot study aimed to measure the compliance of female university athletes with a three-month "Prevent Injury, Enhance Performance" (PEP) warm-up, a validated anterior cruciate ligament (ACL) injury prevention programme, and to gather athletes' perceptions, including barriers to participation in the prevention programme.

Methods

Participants were recruited from "The Ospreys" (Cambridge University's elite sportswomen's club) and considered eligible if they were English-speaking female athletes competing in a high-risk sport, aged 18 to 30. Participants completed a questionnaire pre- and post-completion of three months of the PEP prevention programme. Gathered information included participant demographics, sporting history, education and awareness of ACL injury risk factors, prevention programme perceptions, and compliance.

Results

A total of 45 participants were recruited, with a mean age of 21.69 years (± 2.51). The most common self-perceived risk of ACL injury among participants was 3 (out of 10) on a Likert scale. A total of 62% (n = 28) claimed awareness of ACL injury risk factors. The mean compliance with the prevention programme was 1.46 times per week. The time-consuming nature of the prevention programme was the most reported barrier to compliance (94%, n = 42).

Conclusion

Adherence to a three-month ACL injury prevention programme was limited, and the programme's time-intensive nature was perceived as the most common obstacle to completion. Few athletes were aware of the risk factors and prevention methods for ACL injuries. To prevent the severe long-term impacts of ACL injuries, it is crucial to investigate further the barriers to adopting injury prevention programmes. More time-efficient injury prevention programmes could be developed to improve athlete engagement.

## Introduction

Anterior cruciate ligament (ACL) injuries are among the most common and significant knee ligament injuries, constituting around half of all knee injuries [1]. Due to anatomical, hormonal, neuromuscular, and biomechanical differences (wider pelvis, increased Q angle, and greater ligament laxity), female athletes are at eight to nine times greater risk of ACL rupture than their male counterparts [2-5]. There is also growing evidence to suggest a role for a gendered sporting environment in contributing to the increased risk of female athletes, consisting of disparities in coaching and access to facilities [6]. In female athletes, ACL injuries usually occur in a non-contact setting, with the most common injury mechanism being landing tasks, deceleration, or lateral pivoting that inflicts high external knee joint loads [3].

ACL tears can often be career-ending for an athlete [7]. Indeed, 20% of ACL-injured athletes, both elite and non-elite, do not return to any sport [8]. Those who opt for ACL reconstruction have a mean return-to-play time of 12.2 months. However, even with surgical intervention, there remains a high prevalence of long-term complications, including a one in five risk of re-injury and a high incidence of post-traumatic osteoarthritis, present in approximately 80% of patients at 10 years post-initial injury [9-11].

The annual incidence of ACL injury in the UK is approximately 71 per 100,000 [12]. Around 12,000 ACL reconstructions are performed annually, representing a 12-fold increase over the last two decades [12,13]. Given the ever-increasing female participation in sports [14], the annual ACL reconstruction rate will continue to rise unless preventative measures are taken. ACL injuries represent a physical and emotional burden on the individual and have significant financial consequences for both individuals and society [9].

The catastrophic nature of this injury and increased media coverage of the prevalence of ACL injuries among female athletes have highlighted the need for research to focus on injury prevention [15]. In response, the International Olympic Committee invited a multidisciplinary group of ACL experts to consider the key elements of a successful prevention programme; they concluded that effective programs should be designed as regular warm-up programmes, focusing on strength, power, plyometrics, agility, and neuromuscular training [16]. Additionally, the James Lind Alliance, a non-profit initiative, has identified "What makes an effective tissue knee injury prevention program?" as one of its top 10 research priorities in soft tissue knee injury research [17].

Evidence from a recent meta-analysis showed that prevention programmes can reduce ACL injury risk by 50% in all athletes and 67% for non-contact ACL injuries in females [18]. However, ACL injury rates in women appear to be on the increase despite the availability of such prevention programmes [19]. Barriers might include deficits in athlete and coach education, availability of injury prevention programmes, or poor athlete compliance due to perceived time and financial costs [19]. Indeed, a recent survey study on female university athletes found that 87% of their participants did not engage in ACL injury prevention programmes or perform specific training or exercises aimed at reducing ACL injury risk, even despite a high prevalence of previous ACL injuries among their participants. This survey also suggested a disparity between the participants' perceived risk of ACL injury and the actual risk [20]. Therefore, further evaluation of the efficacy and compliance of athletes with such prevention programmes is required.

The prevention programme selected for this study was the "Prevent Injury, Enhance Performance" (PEP) warm-up, which has been previously demonstrated to significantly reduce athletes' risk of ACL injury [21]. This is designed to be a short warm-up programme to address potential deficits in strength and coordination of stabilising muscles around the knee joint [21].

This pilot study aimed to measure the compliance of university athletes with a three-month PEP programme, a validated injury prevention programme, and to gather athlete perceptions, including barriers to participation.

## Materials and methods

Participants, aged 18 to 29, were recruited. Subjects were considered eligible for the study group if they were female Cambridge University sports athletes participating in a high-risk sport for the duration of the study and English speakers aged 18 to 30. Subjects were recruited from the Ospreys, the University of Cambridge Society for Sportswomen.

Subjects were considered ineligible for participation if they did not speak English, were unable to provide consent, were pregnant or breastfeeding, had arthritis, or had a previous ACL injury. Eligible participants were provided with a Participant Information Sheet (PIS) outlining the study design, objectives, procedures, data management, and dissemination plans. The University of Cambridge Human Biology Research Ethics Committee sought and granted ethical approval (HBREC.2023.17) prior to recruiting any participants for the study. At the start of the assessment, the participants were allowed to ask questions about the study. If they were still interested, the potential participant was asked to complete a consent form and enrolled in the study.

Each participant completed a questionnaire before commencing three months of the PEP warm-up programme [22] (see Supplementary Table 5). This questionnaire was created for this study based on a review of existing literature regarding risk factors for ACL injury. Baseline data recorded before testing included gender, ethnicity, sporting history, previous injury history, past medical history, menstrual history, basic social history, and education and awareness of ACL injury risk factors and prevention programmes. Basic demographic characteristics were also taken, including age, height, and BMI.

This PEP programme is designed to serve as a warm-up prior to a training session or game and is advised to be completed two to three times per week to be effective [22]. It consists of a warm-up, strengthening exercises, plyometrics, agility exercises, and stretching (these can be done before or after training/match). As such, we recommended that participants complete the warm-up two to three times per week consistently for the full three-month period. We provided a list of exercises with specified durations (see Supplementary Table 6) and included video clips to aid in the performance of lesser-known exercises. Participants were asked to record a log of the number of times they performed the PEP programme over the course of the three-month period.

Following three months of the prevention programme, participants completed a second questionnaire, which additionally gathered information on prevention programme compliance and qualitative data regarding participant opinions on the advantages and disadvantages of the prevention programme.

Qualitative data were analysed using thematic analysis, allowing us to identify recurrent themes in the data. This involved data coding initially, where segments of the feedback were identified and assigned descriptive labels that summarised the content. We then developed our themes, allowing the segments to be categorised into broader themes. These themes were then refined to ensure the feedback was accurately represented. Quantitative data were analysed in MS Excel (Microsoft Corporation, Redmond, Washington), in which means and standard deviations were calculated.

Equity, diversity, and inclusion statement

Our research team and author team were gender-balanced and inclusive, comprising four women and four men. Our research team consisted of medical students, junior doctors, consultant doctors, and senior researchers. Our participants were exclusively women, which was to address the under-representation of women in this field of ACL research, in addition to the known increased risk of ACL injury in women compared to men. Our participants were recruited exclusively from the Ospreys (Cambridge University's elite sports society for women); hence, we acknowledge that our findings may not be generalisable to grass-roots levels or in settings with fewer resources.

Patient and public involvement statement

Patients and the public were not involved in the design, recruitment, conduct, or dissemination of the results of this study. This was given a recent James Lind Alliance Delphi Study listed "What makes an effective soft tissue knee injury prevention programme?" as the third highest priority question to be answered. The aforementioned Delphi study involved patients, healthcare professionals, and carers.

## Results

Demographics

A total of 70 participants were recruited; however, only 45 completed phases 1 and 2 of the study and were included in the final dataset. A total of 19 participants were lost to follow-up, and six participants sustained a lower limb injury during the study, resulting in their exclusion. The participants ranged in age from 18 to 29, with an average age of 21.69 years (± 2.51). Participants had a mean weight of 64.36 kg (± 9.15), height of 1.60 m (± 0.06), and BMI of 22.83 kg/m² (± 2.80) (Table 1). Most participants (n = 37) self-identified as being White, with a small minority identifying as being of mixed heritage (n = 6) or Asian heritage (n = 2).

**Table 1:** Participant Demographics The mean age, weight, height, and BMI are presented along with their respective standard deviations (SD).

Variable	Mean (± SD)
Age (years)	21.96 (± 2.51)
Weight (kg)	64.36 (± 9.15)
Height (m)	1.60 (± 0.06)
BMI (kg/m^2^)	22.83 (± 2.80)

Sporting history

Participants came from diverse sporting backgrounds, and many reported participating in multiple sports. The most played sports included rugby (27%), lacrosse (16%), netball (27%), hockey (11%), and football (11%). Table 2 presents a full breakdown of the sports played by the participants. The modal number of sports played by participants was 1 (range: 1-6), and they had played a mode of 12 matches (range: 1-45) this season. Participants varied in their playing history, but on average, they had a long mean playing history of 10.03 years (±5.64). On average, participants had a heavy training schedule, five to six times a week, with a mean number of training sessions of 5.87 (±2.01). Most (98%) reported a strength and conditioning component to their weekly training. Other common training modalities included sport-specific sessions, sports matches, and running/fitness.

**Table 2:** Participants' Sports This is a breakdown of the sports played by the participants. Some participants were represented multiple times if competing at an elite level in multiple sports.

Sport	No. of Participants
Rugby	12
Lacrosse	7
Netball	12
Hockey	5
Football	5
Rowing	4
Athletics	3
Horse Riding	1
Basketball	1
Cheerleading	1
Boxing	1
Swimming	4
Running	3
Australia Rules Football	1
Water polo	2
Badminton	1
Gymnastics	1

In total, 98% of participants (n = 44) reported performing movements at high risk for a non-contact ACL injury, such as quick changes in direction, lateral movements, and jumping. A total of 24% of participants (n = 12) also reported performing high-risk activities for contact ACL injuries, which included tackling and scrummaging. Most (98%) of participants reported performing a warm-up; however, the data were unreliable concerning how frequently participants cooled down after the activity. A total of 98% of participants reported using the appropriate footwear and equipment for their training environment; however, 71% of participants (n = 33) reported at-risk training environment conditions such as wet, icy, slippery, or muddy training surfaces. Table 3 presents a further breakdown of potential participant extrinsic risk factors.

**Table 3:** Training and Equipment-Related Risk Factors A breakdown of potential risk factors and protective factors that could affect the risk of knee injuries for participants.

Risk	Yes	No
Perform strength and conditioning	44	1
At-risk movements for non-contact ACL injury (e.g., quick change of direction, lateral movements, pivoting, jumping)	44	1
At-risk movement for contact ACL injury (e.g. tackling and scrummaging)	11	34
Perform a warm-up	44	1
Appropriate footwear and equipment	44	1
At-risk environment (e.g., muddy, wet, slippery, icy)	33	12
Previous gait analysis	1	44

Perceived risk of ACL injury

**Figure 1:**
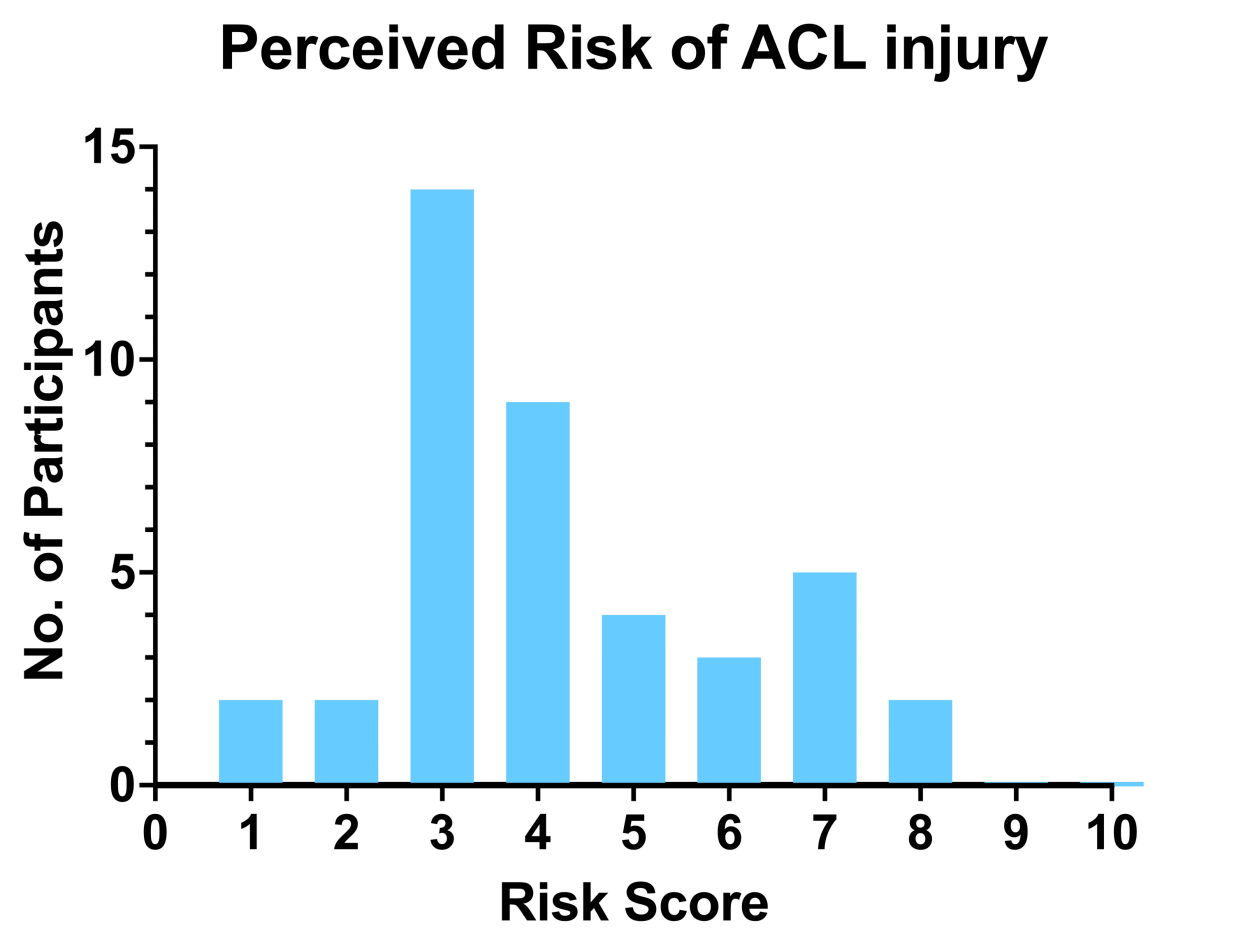
Self-rated perceived risk of ACL injury (0–10 scale) among participants (n = 45). The bar chart displays the perceived risk of ACL injury, as reported by participants, using a Likert scale from 1 to 10, where 10 indicates the highest perceived risk and 1 indicates the lowest perceived risk.

Participants were asked to rate their perceived risk of ACL injury on a 10-point Likert scale, with 1 indicating a low risk of injury and 10 indicating a high risk of injury. The mean Likert score was 4.32 (±1.81), and the mode for self-rated perceived risk was 3 out of 10, as displayed in Figure 1.

Education and awareness

Participants in the study were asked whether they could name any risk factors for soft tissue knee injuries, specifically ACL injuries. Out of 45 participants, 62% (n = 28) reported being aware of risk factors for ACL injuries. However, when directly asked to identify specific risk factors for ACL injury, 75% (n = 34) could identify at least one. When asked to give examples, 21% (n = 10) could name one, 18% (n = 8) could name two, 21% (n = 10) could name three, 14% (n = 6) could name four, and 25% (n = 11) could not name any. Some examples of risk factors provided by participants included female sex, stages of the menstrual cycle, muscle weakness around the knee, sports involving quick changes of direction, family history, and trauma.

Only 13% of the participants had received previous formal education about ACL injuries, risk factors, and prevention techniques. Additionally, only 4% (n = 2) had previously participated in injury prevention programmes.

Prevention programme compliance

**Figure 2:**
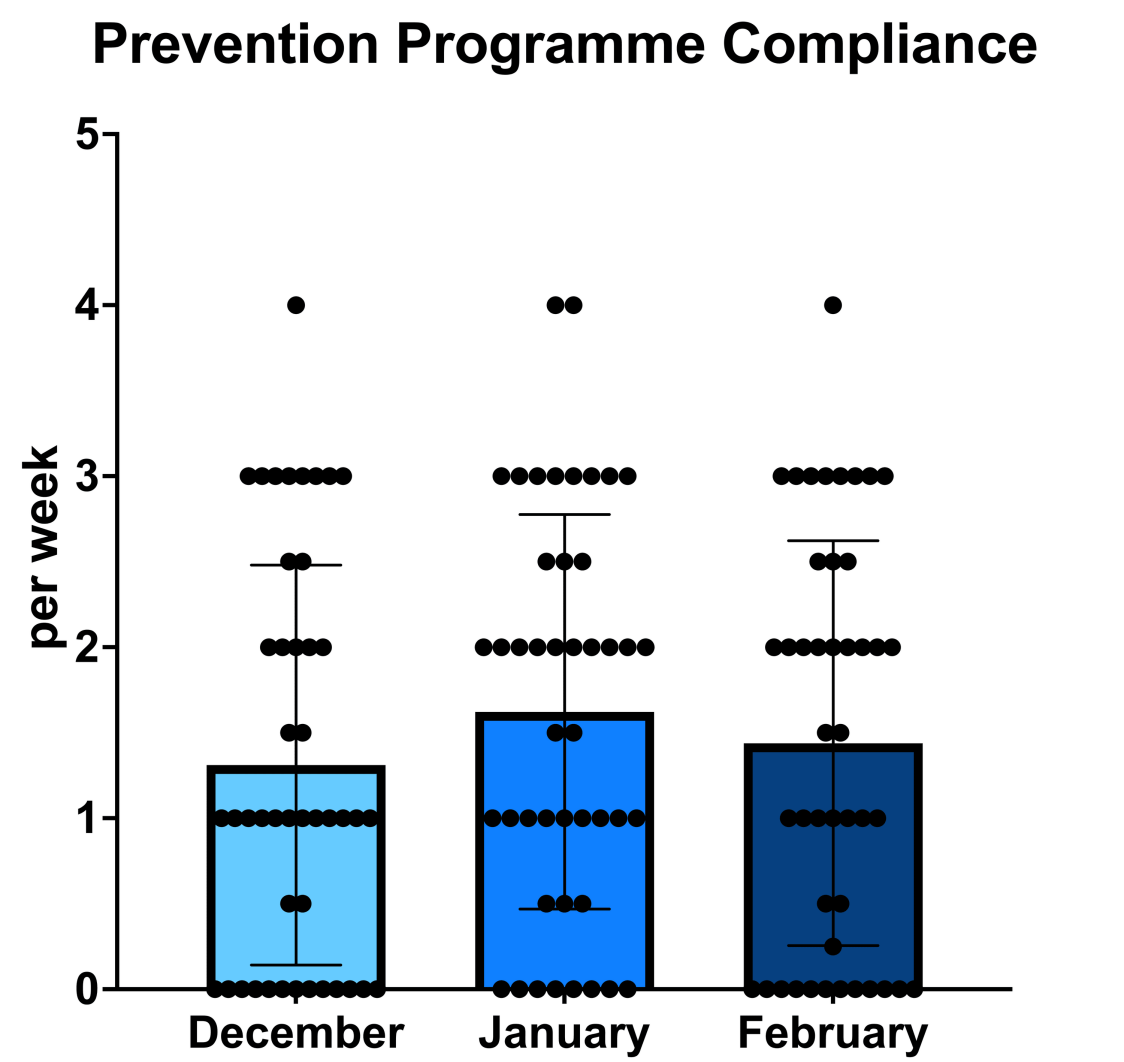
Mean weekly compliance with the PEP warm-up over a three-month period. Data reported via self-assessment in follow-up questionnaires. Bar chart depicting the number of times participants completed the prevention programme per month during the three months. The mean and standard deviation are also plotted.

The mean overall compliance with the prevention programme was 1.46 (±1.17) times per week (see Supplementary Table 4). There was no significant difference in compliance across the three months (p = 0.165) (Figure 2). Seven people (16%) did not attempt the prevention programme at all over the three-month period.

Perceptions of the prevention programme

**Figure 3:**
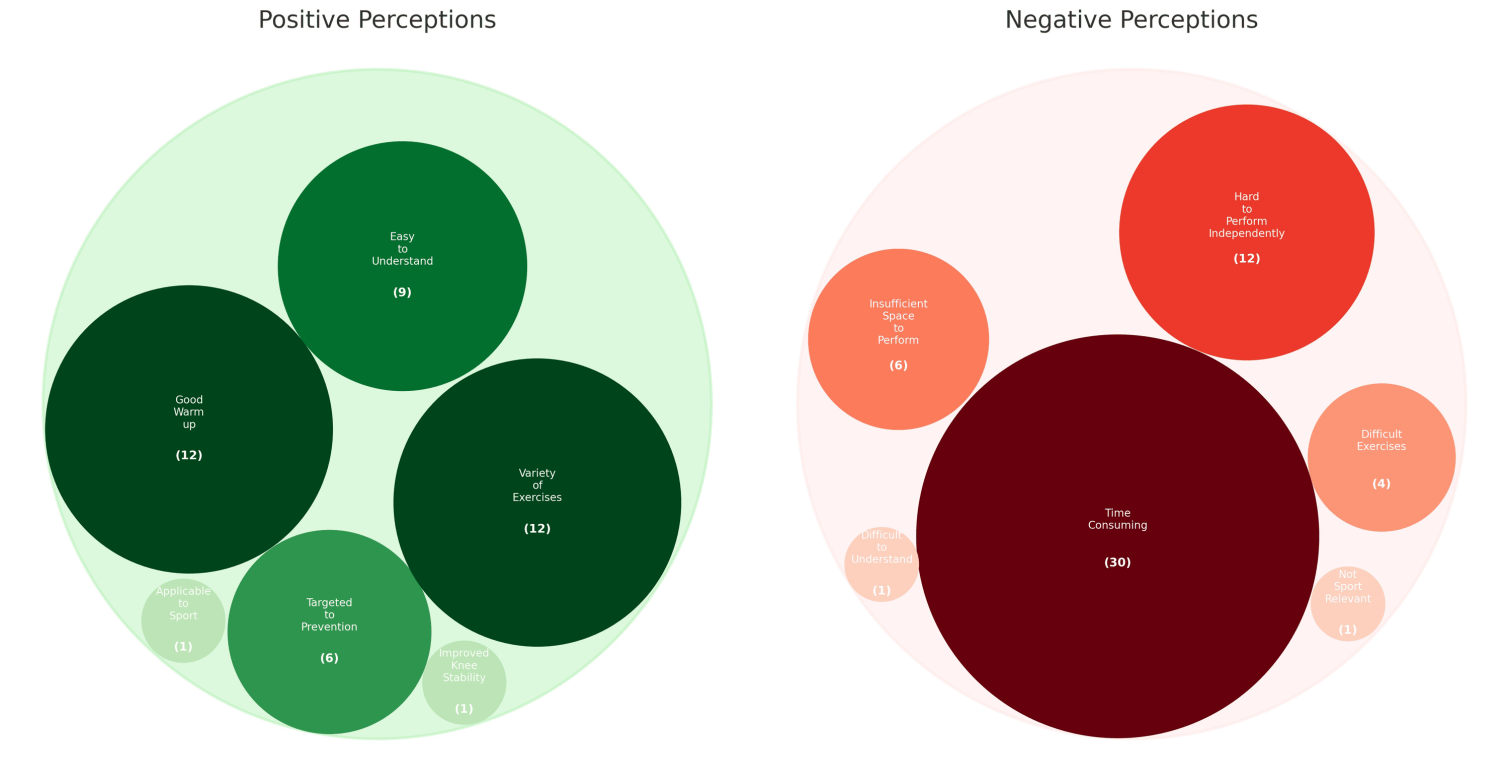
Participant perceptions of the prevention programme Bubble graph illustrating participants' positive and negative feedback after completing the three-month warm-up to the Play ACL injury prevention programme. Bubble size correlates with the frequency of the theme reported. Image created by the authors.

Participants reported both positive and negative perceptions of the prevention programme after the three-month period, along with perceived barriers to completion of the programme (Figure 3).

Many participants reported a thorough warm-up (32%, n = 12) that left them feeling stronger, and they enjoyed the variety of strength, plyometric, and stretching exercises (32%, n = 12), as this introduced new exercises and behaviours they would not otherwise perform. They also reported that the programme was easy to understand and follow, particularly with the video aids (24%, n = 9). Qualitative feedback from participants in sports such as netball, hockey, and football found it easy to incorporate these into their existing training warm-ups, as they were already completing some of the exercises in their warm-ups.

The most common negative feedback and perceived barrier to completing the prevention programme was that it was significantly more time-consuming than anticipated (94%, n = 30). This made it challenging to perform alongside their regular training unless it was incorporated into the whole team warm-up by the captain or coach. Reported compliance was higher in sports, where the warm-up was integrated into the entire team's routine. However, even when incorporated into their training schedule, it remained described as time-consuming. Another key theme was that participants struggled to complete it independently (38%, n = 12), as some exercises required two people, such as Nordic curls. Furthermore, participants expressed a greater need for motivation to complete it alone compared to when performing as part of a team warm-up. Other significant barriers included insufficient space for the running sections of the warm-up (19%, n = 6), the exercises being difficult to perform (19%, n = 6), and the programme itself not being particularly relevant to sports such as swimming and rowing (3%, n = 1).

## Discussion

Participants' self-rated perceived risk was low, with a mode of three (out of 10) on the Likert scale. This is surprising considering participants were recruited from high-risk sports, and many athletes reported that they had been motivated to join the study after teammates had sustained ACL injuries. In total, 62% of participants claimed to be aware of risk factors for ACL injury; however, of this "ACL risk factor aware" cohort, about half (54%) could name one or more specific ACL injury risk factors. This suggests a significant disparity between perceived and actual risks of sustaining an ACL injury. A similar observation was made by Gompels et al. [20] in their pilot survey study of ACL injuries in female university athletes. They found that although 72% of participants believed they were at increased risk of ACL injury compared to males, only 20% identified the risks of developing osteoarthritis. Only 16% recognised the risks of re-injury post-ACL injury. Only 13% of the participants reported having received previous education about ACL injuries, risk factors, and prevention techniques. One could, therefore, speculate that a lack of education may contribute to this disparity in the calculation of athlete injury risk.

Only 4% had previously participated in injury prevention programmes. Similarly, Gompels et al. [20] found that 87% of their participants did not engage in ACL injury prevention programmes or perform specific exercises to reduce the risk of ACL injury.

The athletes in this study had poor compliance with the three-month PEP injury prevention program. They had been instructed to perform the programme at least three times a week and keep a training log. Our athletes performed the programme 1.46 times a week, with seven people not attempting it at all. There was no significant difference in compliance across the three-month period despite university holidays (p = 0.165).

The literature commonly finds poor compliance with injury prevention programmes [23,24]. Indeed, a systematic review of randomised controlled trials of sports injury prevention interventions found that compliance significantly affected study outcomes [24]. Lack of take-up and poor compliance with programmes could explain Webster and Hewett's [18] observation that ACL injury rates appear to increase despite the availability of efficacious injury prevention programmes.

In total, 94% of our participants named the time-consuming nature of the PEP prevention programme as the most significant barrier to compliance. This is consistent with the literature, which has previously reported time consumption as the most significant barrier to prevention programme uptake [19]. Indeed, a survey study on female university athletes found that over half of the participants (51%) would not be willing to invest more than an hour a week in an ACL injury prevention programme [20]. Therefore, one might consider the reasons why it is "time-consuming," given PEP is designed to be a relatively short warm-up; examples might include slower performance due to lack of knowledge or understanding of the exercises or doing it in addition to a sport-based warm-up.

Numerous athletes explained that it was challenging to perform alongside their regular training, as they were required to attend practice early or do a different warm-up separate from the rest of their team. Moreover, reported compliance was improved in individuals who participated in the whole squad's warm-up. This supports previous suggestions that coach education and buy-in are key facilitators to prevention programme uptake [25]. In support of coach education, Ling et al. [26] found that a 60-minute educational workshop on neuromuscular training for coaches, when compared to controls, increased the number of exercises used during the warm-up, the adherence to the neuromuscular training programme and the frequency of alignment cues.

Additionally, 32% of participants reported that performing the programme alone was a barrier due to a lack of motivation or because specific exercises required a partner, such as Nordic curls, which necessitate a partner to perform. Only 19% identified insufficient space for the shuttle/running components of the warm-up in the gym, and 19% found the exercises challenging to complete. Minnig et al. [19] similarly identified these barriers, which support making prevention programmes simple to follow and practical for the training environments available.

Positive perceptions included 32% of participants feeling thoroughly warmed up and stronger before playing and 32% enjoying the variety of strength, plyometric, and stretching exercises. Indeed, Renstrom et al. [16] suggested that programmes that enhance athletic performance and prevent injury could be more likely to be adhered to.

Our pilot study had numerous limitations. Few athletes maintained the training log, so our results are at high risk of recall and reporting bias. Moreover, the athletes were not observed performing the prevention programme, so one cannot assume complete adherence to the PEP exercises. Given that this study relied on volunteer participation and self-directed completion of the PEP programme, the use of a behavioural framework may have strengthened our understanding of factors influencing adherence. Future studies may consider incorporating such frameworks to better explore behavioural barriers to compliance. Additionally, the sample size was small (n = 45) and heterogeneous in terms of the sports played, which raises questions about the validity of our results for the broader sporting population. We experienced a high dropout rate, with 17 participants lost to follow-up and an additional six excluded due to injury. This questions the validity of our results. One can speculate that overall compliance would likely have been even worse if these individuals had been included. It is also necessary to consider the effect of attrition bias in this study, as it is possible that athletes who completed the programme and post-intervention questionnaire could differ systematically from those who dropped out. This may have led to overestimation of average compliance and underestimation of perceived barriers.

Future studies should include larger populations of athletes and examine strategies for addressing the identified barriers. A one-size-fits-all programme is unlikely to be feasible [19]. Examining individual training schedules and behaviours is likely beneficial. For example, 44 out of 45 participants engaged in a gym strength and conditioning session at least once a week, presenting an additional opportunity to implement injury prevention exercises and focus on warm-up regimens.

## Conclusions

This pilot study explored the perspectives of female athletes regarding injury prevention programmes. Only a few athletes knew of ACL injury risk factors and prevention methods. Adherence to a three-month ACL injury prevention programme was limited, and the programme's time-intensive nature was seen as an obstacle to completion. To prevent the severe long-term impacts of these injuries, including post-traumatic osteoarthritis, it is crucial to investigate further the barriers to adopting injury prevention programmes. Furthermore, more time-efficient injury prevention programmes could be developed to better engage athletes.

## Appendices

**Table 4:** Participant Compliance With the Prevention Programme Mean and standard deviation of the times participants completed the prevention programme for each month during the three-month study period and as a three-month average.

Month	Mean (± SD)
December	1.31 (±1.17)/week
January	1.62 (±1.15)/week
February	1.44 (±1.18)/week
3-month average	1.46 (±0.99)/week

**Table 5:** Participant Questionnaire The participant questionnaire was completed before and after the three-month prevention programme. Questions with 'Post-PEP' were included in the post-questionnaire only.

Section	Question	Response Format
Study Info	Study Number	Open-ended
Demographics	Ethnicity	Open-ended
	Age	Open-ended (years)
	Weight	Open-ended (kg)
	Height	Open-ended (cm)
Exclusion Criteria	Current or previous ACL injury?	Yes/No
	Currently pregnant or breastfeeding?	Yes/No
	Inflammatory joint condition (e.g. arthritis)?	Yes/No
	Ongoing lower limb injury?	Yes/No
Sport Participation	What sports do you play and at what level?	Open-ended
	Years of participation in each sport	Open-ended
	Weekly training frequency and type	Open-ended
	Weekly training program description	Open-ended
	Primary sport movements (e.g. cutting, jumping)	Open-ended
	Use of warm-up/cool-down routines?	Yes/No
	Appropriate footwear and equipment used?	Yes/No
	Primary training surface	Open-ended
	Environmental factors affecting training (e.g. surface)	Open-ended
	Previous gait/biomechanical assessment?	Yes/No
	Matches/competitions in the last season* (post-PEP)*	Open-ended
Previous Injury	History of ACL injury	Yes/No
	History of other knee injuries	Yes/No
	History of knee surgery	Yes/No
	Other significant sporting injuries	Yes/No
	History of physiotherapy and reason	Yes/No + Open-ended
Hypermobility	Beighton Score (out of 9)	Numeric input
Medical History	Regular medications	Open-ended
	Musculoskeletal conditions	Open-ended
	Past surgeries	Open-ended
Menstrual/Hormonal History	Willing to answer menstrual cycle questions?	Yes/No
	Have menstrual cycles?	Yes/No
	Length of menstrual cycle	Open-ended
	Current stage of the menstrual cycle	Open-ended
	Use of hormonal contraception (type)	Open-ended
Family History	Family history of early-onset osteoarthritis (<50 years)	Yes/No
Behavioural Factors	Do you smoke? (amount per week)	Open-ended
	Do you drink alcohol? (units per week)	Open-ended
	Use of recreational drugs (type and frequency)	Open-ended
Education & Awareness	Familiarity with ACL risk factors	Yes/No
	List known ACL injury risk factors	Open-ended
	Awareness of ACL prevention strategies	Yes/No
	Received formal education/training on ACL prevention?	Yes/No
	Self-rated ACL injury risk (0–10)	Likert scale (0–10)
Additional Comments	Anything else about athletic background, injury history, or ACL concerns?	Open-ended
Power Up to Play Compliance (post-PEP)	Frequency of programme use in December (per week)	Numeric input
	Frequency of programme use in January (per week)	Numeric input
	Frequency of programme use in February (per week)	Numeric input
	What did you like about the programme?	Open-ended
	What did you not like about the programme?	Open-ended
	Barriers to performing the programme	Open-ended

**Table 6:** Summary of the Components of the PEP Warm-Up A summary of the different components of the PEP warm-up [22].

Component	Exercise	Duration/Reps
1. Warm-up	Jog line to line	30 sec
	Shuttle run (side to side)	30 sec
	Backward running	30 sec
2. Strengthening	Walking lunges	1 min
	Russian hamstring	1 min
	Single toe-raises	1 min
3. Plyometrics	Lateral hops	30 sec
	Forward/backward hops	30 sec
	Single leg hops	30 sec
	Vertical jumps	30 sec
	Scissors jump	30 sec
4. Agilities	Forward run with 3-step deceleration	1 min
	Diagonal runs	1 min
	Bounding run	1 min
5. Stretching (at the end of training/match)	Calf stretch	30 sec × 2 reps each side
	Quadricep stretch	30 sec × 2 reps each side
	Figure 4: hamstring stretch	30 sec × 2 reps each side
	Inner thigh stretch	30 sec × 2 reps each side
	Hip flexor stretch	30 sec × 2 reps each side
